# Eclampsia in a Primipara at the Sixth Month of Utero-Gestation

**Published:** 1860-09

**Authors:** Robert B. S. Hargis

**Affiliations:** Pensacola, Florida


ART. IV.—*Eclampsia in a Primipara at the Sixth Month of Utero-Gestation; Uterine Hemorrhage; Contraction of the Pelvis; Cephalotomy; Metro-Peritonitis; Recovery.* By ROBERT B. S. HARGIS, M.D., of Pensacola, Florida.

ON the 1st of August, 1859, I was hurriedly summoned to Mrs. M., a young woman, aged eighteen years, who was in the sixth month of pregnancy with her first child. According to her husband's account she had been quite healthy until a few weeks ago, when she complained of pain in her loins, and a sense of lassitude, associated with much mental depression and irritability of temper; costiveness, and frequent inclinations and attempts to urinate, without issue, were also troublesome attendants. This morning, while engaged in preparing breakfast, she complained of vertigo, and slight pain and fullness in the head, and on stooping to pick up a fork, she fell upon the floor in a frightful convulsion. I found her in bed writhing in a convulsive throe, which in the course of a few moments abated. Her general appearance indicated poverty of blood, with serous polyæmia; the face, as well as the body and extremities, was œdematous in a remarkable degree; even the eyelids were tensely infiltrated. During the intervals between the convulsive paroxysms she lay upon her back, with her eyes closed, and, excepting respiration, every other function of animal life was entirely suspended; thought, voluntary motion, and sensation were apparently abolished. Respiration was frequent and laborious, without stertor; the iris was contracted and inactive under the influence of light. Hemorrhage had been profuse, but was now moderate; the abdomen was considerably distended, and on a careful examination the gravid uterus could be felt, but no fœtal movements were appreciable; nor were any other indications of a living fœtus in utero manifested by auscultation, fœtal pulsations, or uterine *souffle*. On examination, *per vaginam*, the os uteri was found high up, and looking upward and backward; it was soft and patulous. I introduced the index finger into the opening, and met with the fœtal head presenting at the superior strait. Before withdrawing the hand, I endeavored to insinuate it into the uterus, with the intention of turning and delivering by the feet; but found the antero-posterior diameter of the pelvis so contracted that three fingers could scarcely be pushed above the brim. I immediately desisted, and sent for a *confrère*, who, after making a careful examination, agreed, without the slightest hesitation, as to the propriety of delivering immediately by craniotomy. This procedure was effected with considerable difficulty, with the perforator and crotchet, in a few moments, and from the exceeding great care observed in its performance, but little injury, if indeed any, was inflicted upon the pelvic organs of the patient. The placenta having been detached, no doubt during the first convulsive throes, came away with the fœtus entire. The convulsions ceased immediately after delivery, and considerable hemorrhage occurred, but was arrested in a few seconds. Respiration was now short and frequent; pulse 160 per minute, small and feeble; skin cool and moist; insensibility perfect. Sinapisms were applied to the spine and epigastrium, and a few drops of spirit of nitric ether, in camphor mixture, given, and ordered to be repeated every twenty minutes, till warmth should be restored to the skin. In the course of an hour and a half considerable reaction ensued, and a purgative enema, consisting of olive oil, castor oil, and infusion of flaxseed, was administered, which brought away an immense quantity of exceedingly offensive fæces, accompanied by a very scanty flow of turbid urine. The patient was now left in charge of a nurse, who was instructed to give an infusion of pennyroyal leaves, with a few drops of sweet spirits of nitre occasionally, and now and then a little water.

August 2.—Mrs. M. is still deprived of the power of feeling or perceiving; lies, as it were, in a profound slumber; has not moved since seen yesterday; skin very warm and moist; pulse 140 per minute, and somewhat fuller and stronger than last evening. A small quantity of urine that had passed during the night was preserved for examination this morning; it was perfectly free from deposits, and on application of the usual tests for albumen, the presence of a considerable quantity of that substance was apparent. Attributing the cause of the coma to urea or some other excrementitious substance that should have been eliminated by the kidneys, and satisfied that those organs were in a congested or hyperæmic condition, I ordered six wet cups to be applied over them, and the following purgative to be given immediately:—

℞.—Hydrarg. chlor. mit, grs. x;
Pulv. rhei., grs. xij.
M.

In the evening she was still insensible; bowels freely moved; urine had dribbled from the meatus pretty freely; pulse 156; skin moist, and very warm.

℞.—Hydrarg. chlor. mit, grs. viij;
Cretæ preparat, grs. xviij.
M. et ft. chart. vi. s.

One every three hours in syrup, with tinct. verat. virid. gtt. iij; re-apply cups to the lumbar region, and put emplast. vesicat., 2 by 6, to the nape of the neck.

August 3. A.M.—Aroused voluntarily at four o'clock this morning, and asked for coffee, and said she felt very badly; œdema of the eyelids and face has measurably disappeared; her legs also are much less swollen; complains of inability to move or draw up the inferior extremities without more or less pain within the abdomen; has a cough; tongue covered, posteriorily, with a dark-brown fur; pulse 118; abdomen tender and tympanitic; bowels somewhat irritable; had several small, greenish stools; urinated freely this morning; has slight discharge from the vagina, which is very dark and offensive. Suspend hydrarg. chlor. mit. and other remedies.

℞.—Potass. Chlorat, ʒij;

Aquæ fontan, ℥viij;

Tinct. verat. virid, gtt. xx.

M. One tablespoonful every third hour in some infusion of flaxseed; and inject into the vagina, occasionally, two or three syringesful of the following:—

℞.—Sodæ chlorinatæ, ℥ss;

Aquæ destillat, ℥xv.

P.M.—Has coughed a good deal during the day; has pain in the lumbar region; skin warm and moist; pulse 120; no motion of the bowels since morning; urinated well. Continue treatment.

August 4. A.M.—Had a restless night; coughed frequently; pulse 116; tongue brown; thirst; abdomen still painful on firm pressure, and tympanitic; bowels open; urination not so free; discharges from vagina increased in quantity, and less offensive.

℞.—Hydrarg. chlor. mit, grs. ix;
Pulv. ipecac. et opii, grs. xlviij.

M. Ft. pulv., No. 6, one alternating with the mixture of potassa and veratrum viride, at intervals of an hour and a half. Apply a cloth wrung out of equal parts ol. terebinthinæ et ol. olivæ.

P.M.—Has a better appearance; had some repose; feels refreshed; skin moist and warm; pulse 108; complains of nausea occasionally; tongue moist; less thirst; pain on pressure of the abdomen not so exquisite; less tympanites; has taken some light nourishment, but did not relish it.

August 5, A.M.—Says she feels better; vomited once in the night, and therefore, according to instructions, one dose of the chlorate and verat. virid. mixture was omitted; bowels acted upon twice during the night, stools feculent and colored with bile; has considerable cough, and pain in the chest; expectoration easy, but rather glairy and tenacious; skin cool and moist; pulse 108; discharging from the vagina freely; abdomen not so sensitive and not nearly so tympanitic. Continue chlorate mixture and powders.

P.M.—No apparent difference in her condition generally. She, however, micturates freely, and begs to be relieved of her cough. Increase the quantity of pulv. ipecac. et opii to grs. x, and apply emp. vesicat. to the chest.

August 6, A.M.—Responds cheerfully to the morning salutation, and says she feels much better; rested well last night; cough less troublesome; pain in the chest relieved; skin cool; pulse 108; slight pain on pressure of the abdomen; gaseous distention nearly absent; can flex the thighs toward the body with very little complaint; desires no food, but does not object to taking some broth. Suspend the calomel, and continue chlorate mixture and Dover's powder.

August 7.—General aspect infinitely better; cough abated; discharge from vagina moderate, and free from fetor; tongue cleaning; was inclined to vomit during the night; had not taken a dose of the chlorate mixture for six hours; pulse 94; desires food. Continue the mixture in smaller doses, and let her have a Dover's powder at night, if not disposed to rest well, or if cough is troublesome.

August 8.—Greets me cheerfully; rested well after taking the Dover's powder; skin quite natural; tongue clean; can move the limbs freely without pain; coughs occasionally; expectorates a little at a time and easily. Prescribed infus. anthemidis, a wineglassful three or four times a day, and tinct. opii camph., with syrup scillæ for her cough.

August 9.—Perspired freely last night; looks pale; complains of weakness; coughs every now and then; bowels open; urine free; desires food; is allowed boiled chicken, with a small quantity of port wine at meal times. Continue infus. anthemid., and tinct. opii camph. to allay cough.

August 11.—Says she felt pretty well yesterday; is sitting up; has been changing her clothes; looks pallid and fatigued; respiration hurried and loud; pulse 120 and small; tongue clean, but anæmic; appetite good; remarked that she did not know how weak she was till she got up to arrange her dress; did not complain of anything else but debility. Her bowels were in a good state, and appetite keen.

I waited about half an hour after she resumed the recumbent position, and found her pulse had fallen to 90, her respirations being very little more frequent than natural. On application of the stethoscope over the cardiac region, venous murmurs were clearly appreciable; and along the course of the large veins of the neck a venous hum was quite audible. "Here," I thought, Professor Meigs would say, "is a case in which the organs are imperfectly innervated, while the patient is in the act of taking exercise from want of power to supply the brain with the normal quantity of oxygenated blood. She has too little oxygen, and the disease is (*now*) debility of her endangium." I would not affect to agree with the learned professor entirely. Nevertheless upon this hypothesis I prescribed—

℞.—Potassæ chlorat, ʒij;

Tinct. ferri. chloridi, ʒiij;

Aquæ destillat, ℥viij.

M. One tablespoonful three times a day.

Under this treatment, with a pill of aloes occasionally, Mrs. M. gradually improved, and in ten days recovered sufficiently to resume her ordinary duties.

